# WHO fact sheet on infertility gives hope to millions of infertile couples worldwide

**Published:** 2020-01-08

**Authors:** Willem Ombelet

**Affiliations:** Genk Institute for Fertility Technology, ZOL Hospitals, Schiepse Bos 6, 3600 Genk, Belgium.

**Keywords:** equity, fact sheet, infertility, low and middle income countries, LMIC, reproductive rights, universal access, WHO

## Abstract

Although the consequences of infertility are often severe in LMIC (Low and Middle Income Countries), national and international health strategies have always focussed on reducing total fertility rates while infertility care has received no attention at all. Access to infertility care can only be achieved when good quality but affordable infertility care is linked to more effective family planning programmes. Only a global project with respect to socio-cultural, ethical, economical and political differences can be successful. The 2020 WHO fact sheet on infertility cannot be misunderstood; global access to high-quality services for family planning including fertility care is one of the core elements of reproductive health. Different strategies are presented.

Infertility is a universal health issue and it has been estimated that 8 to 12% of the couples worldwide are in- fertile (Boivin et al., 2007). Estimates suggest that between 48 million couples and 186 million individuals live with infertility globally, half of these couples are living in Sub-Saharan Africa (SSA) and South Asia (Rutstein and Shah, 2004; Mascarenhas et al., 2012).

Consequences of involuntary childlessness are usually more dramatic in LMIC (Low and Middle Income Countries) when compared to Western societies, particularly for women (Van Balen and Bos, 2009; Ombelet et al., 2008; Ombelet, 2011; Inhorn and Patrizio, 2015). Childless women are frequently stigmatised, isolated, disinherited and neglected by the entire family and the local community. This may result in physical and psy- chological violence, polygamy, even suicide. Because many families in LMIC completely depend on children for economic survival, childlessness has to be regarded as a social and public health issue and not only as an individual medical problem (Dyer et al, 2012).

Despite its relatively high prevalence and the cultural values associated with childbearing, infertility care remains a low priority area for local health care providers and community leaders, not only on a national but also on an international level.

This can be explained by two commonly used arguments; the “limited resources” and the “overpopulation” argument.

The lack of infertility treatment services is mostly justified as a form of population control, particularly in high-fertility settings such as SSA and Asia. Infertility may be used as a ‘solution to overpopulation’ and as a ‘low-priority issue’ in the context of scarce health care resources, poor medical infrastructure, and the heavy burden of other life-threatening problems such as HIV/AIDS, malaria, tuberculosis and maternal mortality (Inhorn and Patrizio, 2015).

The world population is expected to increase by 2 billion people in the next 30 years, from 7.7 billion currently to 9.7 billion in 2050 and could peak at nearly 11 billion around 2100 (United Nations, 2019). The argument of overpopulation suggests that in countries where overpopulation poses a demographic problem, infertility management should not be supported by the government.

Many LMIC already succeeded to drop their global fertility rate (number of children per woman) and United Nations data clearly show that in the majority of LMIC the mean fertility rate is expected to decline below the replacement level of 2.1 by mid-century with the exception of Sub-Saharan Africa.

UN figures also show that an improved life expectancy will be the most important factor considering world population growth. Even in the least developed countries life expectancy is going to rise from an average of 51.1 in 1990, 65.2 in 2019 to 71.8 years in 2050 which highlights the important issue of population ageing (United Nations, 2019).

Even if infertility treatment could be made more accessible in LMIC it would probably account for less than 1 % of all deliveries. Increasing efforts on family planning and health education should readily overcome this small contribution to the fertility rate.

Nevertheless, the idea of infertility treatment in developing countries often evokes a feeling of discomfort and disbelief. Subsequently national and international health strategies have always focussed on reducing total fertility rates while infertility care has received no attention at all. This narrow approach contradicts human rights in general and reproductive rights in particular.

A recent systematic landscape analysis showed that in LMIC no studies could be found reporting the imple- mentation of low-cost assisted reproductive technology (ART) being effective, affordable and accessible to most in need of the services (Chiware et al., 2020).

Nevertheless, promising low cost in vitro fertilisation (IVF) techniques are being developed with promising results (Van Blerkom et al., 2014). Low-cost screening tests for infections can be used, a one-day diagnostic approach seems to be feasible (Ombelet and Campo, 2008), high-quality automated Smartphone-based semen analysis systems are available (Kobori, 2019) and low-cost mild ovarian stimulation protocols can be used with good results (Nargund et al., 2017). Reasons enough to start actions to increase universal access to infertility care.

## International statements

60 years ago, at the 1948 UN Universal Declaration of Human Rights, the following statement was adopted: “Men and woman of full age, without any limitation due to race, nationality or religion, have the right to marry and to raise a family”. This statement implies the right to access to fertility treatments when couples are unable to have children. According to the World Health Organisation (WHO) “Reproductive health addresses the reproductive processes, functions and system at all stages of life. Reproductive health, therefore, implies that people are able to have a responsible, satisfying and safe sex life and that they have the capability to reproduce and the freedom to decide if, when and how often to do”.

Many years later, at the United Nations International Conference on Population and Development in Cairo in 1994, the final declaration mentioned that “Reproductive health therefore implies that people have the capability to reproduce and the freedom to decide if, when and how often to do so ... and to have the information and the means to do so ...”. In 2004 the World Health Assembly proposed five core statements, including “the provision of high-quality services for family-planning, including infertility services” (World Health Assembly, 2004; UNFPA, 2005). This declaration was signed by many LMIC. Nevertheless infertility care was not mentioned in the Millennium Development Goal five (MDG5) although achieving universal access to reproductive health was one of the main targets. MDG5 mainly focussed on increasing contraceptive prevalence rate and antenatal care coverage.

On September 14, 2020, the WHO published a fact sheet on infertility. The WHO recognises that the provision of high-quality services for family-planning, including fertility care services, is one of the core elements of reproductive health. This very important message includes the wish to do research on global etiological and epidemiological research, facilitating policy dialogue with countries worldwide on infertility care, developing guidelines on the prevention, diagnosis and treatment of male and female infertility, collaborating with relevant stakeholders to deliver fertility care globally and last but not least providing country-level technical support to member states to develop or strengthen implementation of national fertility policies and services (WHO fact sheet on infertility, 2020).

It is clear that affordable solutions for universal infertility care must become operational. Time has come to give equitable access to effective and safe infertility care in LMIC. I sincerely hope that actions will follow very soon and that infertility care will become integrated into mainstream reproductive health care in LMIC. This achievement has the potential to give dignity not only to millions of infertile couples but also to reproductive health care programs and organisations. Family planning cannot be complete without taking care of the infertile and childless population.
